# Practical considerations for the use of fenfluramine to manage patients with Dravet syndrome or Lennox–Gastaut syndrome in clinical practice

**DOI:** 10.1002/epi4.12998

**Published:** 2024-07-04

**Authors:** Elaine C. Wirrell, Lieven Lagae, Ingrid E. Scheffer, J. Helen Cross, Nicola Specchio, Adam Strzelczyk

**Affiliations:** ^1^ Divisions of Child and Adolescent Neurology and Epilepsy, Department of Neurology Mayo Clinic Rochester Minnesota USA; ^2^ Member of the European Reference Network EpiCARE, Department of Pediatric Neurology University of Leuven Leuven Belgium; ^3^ Austin Hospital and Royal Children’'s Hospital, Florey and Murdoch Children's Research Institutes University of Melbourne Melbourne Victoria Australia; ^4^ Developmental Neurosciences Research & Teaching Department UCL NIHR BRC Great Ormond Street Institute of Child Health London UK; ^5^ Department of Neurology Great Ormond Street Hospital London UK; ^6^ Neurology, Epilepsy and Movement Disorders Unit Bambino Gesù Children's Hospital, IRCCS, Full Member of European Reference Network EpiCARE Rome Italy; ^7^ Goethe‐University Frankfurt, Epilepsy Center Frankfurt Rhine‐Main and Department of Neurology University Hospital Frankfurt Frankfurt am Main Germany

**Keywords:** antiseizure medications, Dravet syndrome, fenfluramine, Lennox–Gastaut syndrome, polytherapy

## Abstract

Fenfluramine (FFA), an antiseizure medication (ASM) with serotonergic and sigma‐1 receptor activity, is used to manage patients with developmental and epileptic encephalopathies (DEEs). It is approved in the US for treating seizures associated with Dravet syndrome (DS) and Lennox–Gastaut syndrome (LGS) in patients ≥2 years old and as add‐on therapy for seizures associated with DS and LGS in the EU, UK, and Japan in similarly aged patients. Consensus guidelines for treatment of DS have recommended FFA to be an early‐line ASM, and it has also shown efficacy in managing seizures associated with LGS. DS and LGS are DEEs associated with a range of seizure types, developmental impairments, and multiple comorbidities. Here we provide case vignettes describing 4 patients (3 DS and 1 LGS) aged 4–29 years old in whom up to 14 ASMs had previously failed, to illustrate real‐world practice issues encountered by neurologists. This review provides guidance on the use of FFA in the context of ASM polytherapy and drug–drug interactions (DDIs), behavioral issues, dose titration, and adverse events. Along with data from the clinical trial program, these case vignettes emphasize the low risk of DDIs, a generally well‐tolerated safety profile, and other seizure and nonseizure benefits (eg, improved cognition and sleep) associated with the use of FFA in DS or LGS.

**Plain Language Summary:**

Fenfluramine is used to treat seizures in individuals with Dravet syndrome and Lennox–Gastaut syndrome, but there are a range of issues that clinicians may face when treating patients. This review highlights four patients from the authors’ everyday clinical work and offers guidance and practical considerations by neurologists with expertise in managing these complex conditions related to drug interactions, dosing, and side effects associated with fenfluramine.

## Graphical abstract

This is a graphical representation of the abstract and article highlights.

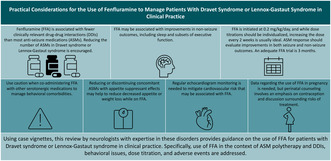

**Key points**

- Fenfluramine (FFA) is an antiseizure medication (ASM) used in the treatment of seizures associated with Dravet syndrome (DS) and Lennox–Gastaut syndrome (LGS).
- FFA is initiated at 0.2 mg/kg/day but speed of titration may depend on age, weight, and concomitant medications.
- FFA is generally well‐tolerated and associated with a low risk of clinically relevant drug–drug interactions, which are often observed with polypharmacy.
- Nonseizure benefits are observed in addition to reduction in seizures, and may include improved sleep and behavior.

## 1 INTRODUCTION

Dravet syndrome (DS) and Lennox–Gastaut syndrome (LGS) are both rare developmental and epileptic encephalopathies (DEEs) characterized by drug‐resistant seizures of multiple types.^1^, ^2^, ^3^, ^4^ Developmental impairment is a key component of a DEE and occurs secondary to the underlying etiology and the epileptiform activity.^1^, ^5^, ^6^ In 90% of cases, DS is associated with a pathogenic variant in the gene that encodes the α1 subunit of the sodium channel, *SCN1A*.^3^, ^7^, ^8^ In contrast, LGS is highly heterogeneous in terms of etiology,^9^ often evolves from another epilepsy syndrome,^2^ and, in approximately 25% of cases, the etiology is unknown.^10^ In both syndromes, seizures persist into adulthood and seizure freedom is rare. The overall treatment goal is to improve patient and caregiver quality of life (QOL) by optimizing use of antiseizure medication (ASM), which includes reduction in seizure burden and improving nonseizure outcomes, as well as minimizing adverse events (AEs).^3^, ^11^, ^12^, ^13^, ^14^ A tailored approach incorporating patient‐specific goals should be developed by clinicians and patient and caregivers.^10^, ^13^

**FIGURE 1:**
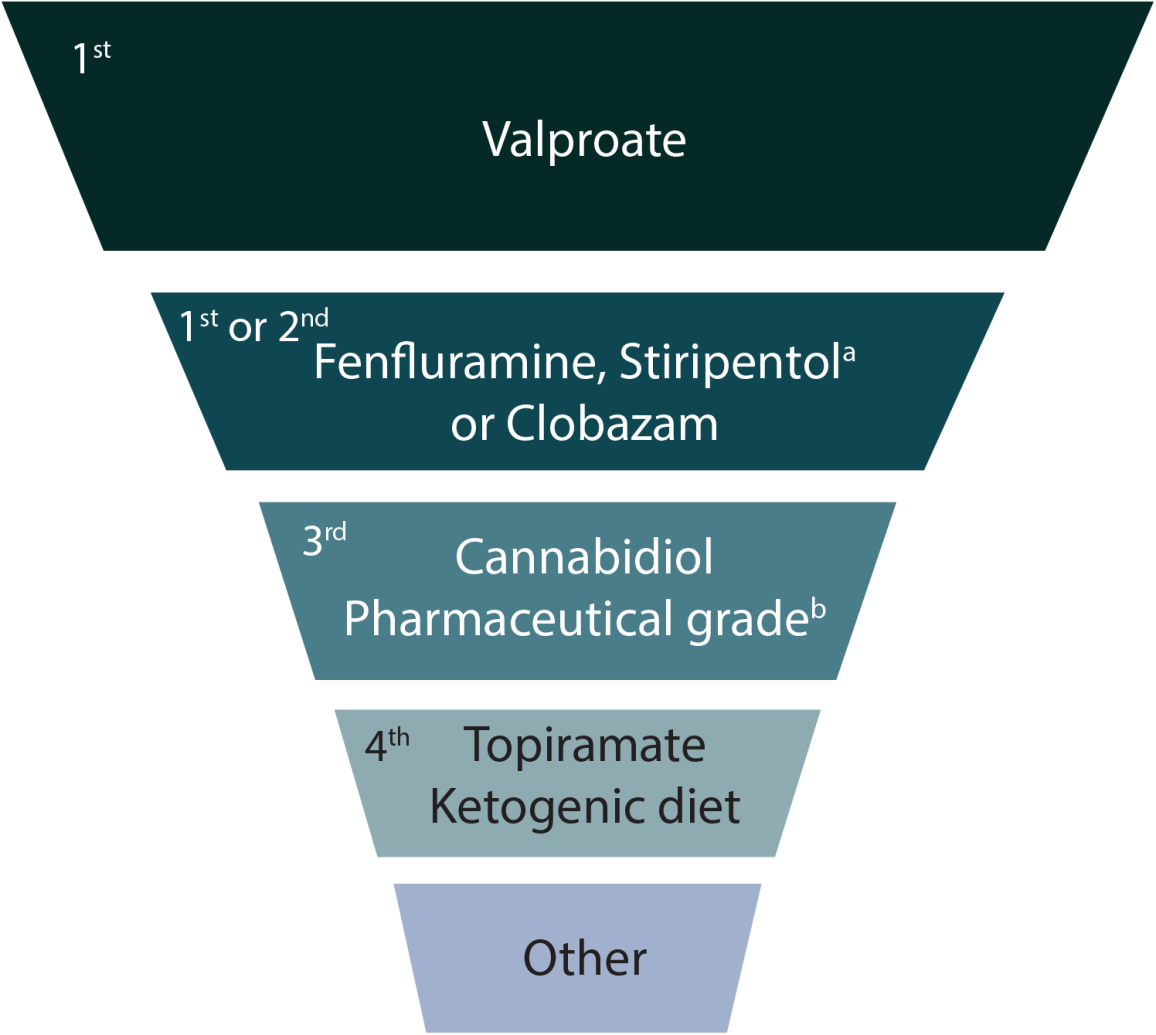
Lines of therapy for treatment of DS. Adapted from Wirrell EC, et al. *Epilepsia*. 2022;63 (7):1761–77, with permission from John Wiley and Sons.^3^ ^a^No data to support monotherapy in DS; in EU, must be co‐administered with CLB and VPA. ^b^In EU, must be co‐administered with CLB. CLB, clobazam; DS, Dravet syndrome; VPA, valproate.

Polypharmacy is usual in patients with DEEs. Patients with DS and LGS are on a median of 3 ASMs at any given time,^3^, ^15^, ^16^, ^17^, ^18^ which does not include therapies for other comorbidities. As seizures are drug resistant, patients cycle through a range of ASMs in their lifetime.^15^, ^19^ An International DS Consensus recently published recommendations for management of DS suggesting valproate (VPA) as the first‐line ASM; additional ASMs can be used concomitantly as first‐ or second‐line options (Figure 1).^3^ While the choice of the most appropriate ASM regimen in LGS is also complex, expert opinion recommends VPA as first‐line followed by lamotrigine (LTG)^20^; for subsequent lines of therapy, there are various ASMs approved for use in the US and EU (Table 1)^10^, ^21^ without clear guidance to sequence treatment selection. It should be noted that the use of VPA as the first‐line agent in both DS and LGS is based on clinical experience, cost‐effectiveness, and positive impact on some comorbidities.^10^, ^22^ It is not licensed for use in DS or LGS, has only been evaluated in uncontrolled studies, and is associated with some rare, but serious, AEs (hepatotoxicity, pancreatitis, and coagulation disorders).^10^, ^23^, ^24^

**TABLE 1:** ASMs approved by regulatory authorities for the management of seizures in LGS in the US or the EU.

ASM	Indicated for the management of seizures (monotherapy or adjunct) in LGS	
	The US	Europe
Valproate	—	—
Lamotrigine	**√**	**√**
Rufinamide	**√**	**√**
Fenfluramine	**√**	**√**
Cannabidiol	**√**	**√** ^a^
Topiramate	**√**	**√**
Clonazepam	**√**	**√**
Felbamate	**√**	**√** ^b^
Clobazam	**√**	—
Perampanel	—	—
Zonisamide	—	—
Levetiracetam	—	—
Lacosamide	—	—
Brivaracetam	—	—
Cenobamate	—	—

*Note*: Adapted from Riva 2022 Seizure^4^; Strzelczyk 2021 CNS Drugs^10^; Montouris 2020 Epilepsy Behav^22^; Besag 2021 Pediatr Drugs.^73^

Abbreviations: ASMs, antiseizure medications; LGS, Lennox–Gastaut syndrome.

^a^ In conjunction with clobazam.

^b^ Felbamate is licensed for adjunctive treatment of seizures in LGS in some European countries.

Fenfluramine (FFA) is the latest ASM to achieve approval for the management of seizures associated with DS and LGS in the US in patients ≥2 years old,^25^ and as add‐on treatment for patients ≥2 years old with seizures associated with DS and LGS in the EU, UK, and Japan.^26^, ^27^, ^28^, ^29^ FFA exerts its antiseizure effects through serotonergic activity and a positive sigma‐1 receptor modulation mechanism.^11^, ^30^, ^31^, ^32^ FFA has demonstrated both short‐ and long‐term seizure efficacy in DS and LGS patient populations^16^, ^33^, ^34^, ^35^, ^36^, ^37^, ^38^ and has demonstrated particular efficacy in reducing generalized tonic–clonic seizure (GTCS) frequency from baseline in both DS and LGS.^33^, ^39^ Thus, FFA is recommended to be a first‐ or second‐line treatment option in DS (Figure 1); updated LGS consensus guidelines are yet to be developed.

In DS, eight reviews have provided indirect comparisons of FFA versus other ASMs, including cannabidiol (CBD) and stiripentol (STP). In the absence of head‐to‐head studies, FFA has provided more effective convulsive seizure control than CBD in patients with DS.^40^, ^41^, ^42^, ^43^, ^44^, ^45^, ^46^, ^47^ Some reviews have reported comparable efficacy between FFA and STP,^40^, ^42^, ^43^, ^44^, ^47^ but interpretation may be limited due to the STP randomized controlled trials (RCTs) having different study designs and methodology, including small sample sizes.^48^, ^49^, ^50^, ^51^ From evaluation of the DS open‐label extension (OLE) data, one network meta‐analysis concluded that the order of probability of reducing seizure frequency by ≥50% was: FFA (effect size [ES]: 0.715, 95% CI: 0.621–0.808), STP (ES: 0.604, 95% CI: 0.502–0.706), and then CBD (ES: 0.448, 95% CI: 0.403–0.493).^46^ When evaluating safety of ASMs for DS, FFA is better tolerated than STP and is generally well‐tolerated compared with CBD^40^, ^52^; less frequent serious TEAEs have been reported with FFA versus both CBD and STP.^40^, ^41^, ^46^ Another network meta‐analysis evaluated six ASMs for adjunctive treatment of various epilepsies and reported that CBD was the most effective ASM for LGS, but also highlighted that, since patients require polypharmacy, FFA and CBD are important new treatment options for LGS.^45^

These literature reviews support the results of the FFA clinical trial program, specifically sustained efficacy in seizure reduction and a tolerable safety profile.^16^, ^33^, ^34^, ^35^, ^37^, ^53^ In this review, we use case vignettes to illustrate real‐world issues experienced by expert clinicians and offer practical guidance on the use of FFA in managing patients with DS and LGS.

## 2 CASE VIGNETTE OVERVIEW

A team of experts who manage patients with complex epilepsies discussed key clinical learnings surrounding the use of adjunctive FFA in three patients with DS and one patient with LGS (Table 2).

**TABLE 2:** Case vignette overview.

	Case 1	Case 2	Case 3	Case 4
Pertinent History				
Age at seizure onset (months)	4	10	12	48
Genetic variant	+*SCN1A*	+*SCN1A*	+*SCN1A*	Ring chromosome 20 syndrome
Age at DEE diagnosis, DEE	13 months, DS	3 years, LGS (diagnosis changed to DS as adult)	4 years, DS	4 years, LGS
Seizure types experienced	Focal GTCS	FIAS GTCS	Seizures associated with fall^a^ Myoclonic GTCS Atypical absence	Tonic Atonic/seizures associated with fall^a^ Atypical absence Absence
Behavioral issues	None reported	Autistic features	Aggression, post‐ictal agitation, restlessness, irritability, impulse control disorder	Intermittent autistic features
Issues with sleep	None	None	None	Intermittent sleep issues due to frequent tonic seizures
Prior antiseizure therapies	LEV, TPM, CBD, ketogenic diet	Bromide, LEV, OXC, PER, LCS, TPM, ZNS, BRV, RUF, VNS, PHB, CZP, STP, CBD	PHB, CBZ, VGB, LTG, TPM, LEV	CBZ, LEV, SUL, ELF, GBP, LTG, CLB, VNS
Demographics				
Sex	Male	Male	Male	Female
Current age (years)	4	29	20	16
Current weight (kg)	12.9	88	95	53
Current Treatment				
FFA regimen	0.35 mg/kg/day	0.25 mg/kg/day	0.19 mg/kg/day	0.29 mg/kg/day
Concomitant antiseizure therapies	VPA, STP, CLB	VPA, CLB	VPA, STP, CLB	VPA, OXC
Other relevant medication notes	VPA and CLB were within therapeutic range when FFA initiated	STP discontinued and CLB dose increased when FFA initiated	On risperidone before FFA initiated	LEV discontinued due to behavioral AEs, all other previous ASMs discontinued due to lack of efficacy
FFA treatment duration	46 months	21 months	12 months	7+ years
Overall Response After FFA				
Seizure Outcomes	Decrease in seizure frequency from weekly convulsive seizures and the use of monthly rescue to 1–2 convulsive seizures bimonthly, no use of rescue	2 seizures/mo (GTCS or FIAS), stable from before starting FFA; no GTCS since treatment with FFA	50% reduction in frequency of all seizure types (from 3–4/year to 1–2/year)	No seizures associated with fall
Nonseizure outcomes	Not observed	Not observed	Improvement in behavior after FFA initiation and switch from risperidone to aripiprazole	Improvement in sleep and behavior after FFA initiation (likely due to improved seizure control)
Caregiver or Patient Perspectives	FFA has been most effective ASM thus far	Improved overall state of health		Caregivers happy with no daytime seizure activity
Adverse Events (AEs)				
Related to FFA	Decreased appetite, weight decreased	None	None	None
Other AEs	None	Diarrhea resolved after discontinuation of CBD	AEs secondary to other ASMs resolved (hyponatremia, acne, incontinence)	None

Abbreviations: AEs, adverse events; ASMs, antiseizure medications; BRV, brivaracetam; CBD, cannabidiol; CBZ, carbamazepine; CLB, clobazam; CZP, clonazepam; DEE, developmental and epileptic encephalopathy; ELF, ethyl loflazepate; FFA, fenfluramine; FIAS, focal impaired awareness seizures; GBP, gabapentin; GTCS, generalized tonic–clonic seizures; LCS, lacosamide; LEV, levetiracetam; LTG, lamotrigine; OXC, oxcarbazepine; PER, perampanel; PHB, phenobarbital; RUF, rufinamide; STP, stiripentol; SUL, sulthiame; TPM, topiramate; VGB, vigabatrin; VNS, vagus nerve stimulation; VPA, valproate; ZNS, zonisamide.

^a^ Also known as drop seizures or drop attacks.

Of the three patients with DS, two were diagnosed when young and one adult received a clinical diagnosis of LGS in early childhood, but the diagnosis was later changed to DS based on reviewing clinical features and genetic etiology (Case 2). Cases 1, 2, and 3 harbored an *SCN1A* pathogenic variant and Case 4 (LGS, previously described^54^) has ring chromosome 20. All developed seizures by 4 years of age and had previously tried 3–14 ASMs. Three patients had significant behavioral issues prior to initiating FFA (Table 2).

Among the four patients (3 male, 1 female), two initiated FFA as children (4 and 9 years old) and two as adults (20 and 27 years old). All patients remain on FFA, and all are on VPA. Two patients with DS are on STP with CLB. Case 3 reduced VPA dose due to hyponatremia, and Case 2 discontinued STP when FFA was initiated.

Throughout an FFA treatment duration of up to 7 years, clinicians managing these four patients encountered a variety of practical issues including, but not limited to, the management of drug–drug interactions (DDIs) with concomitant ASMs, pharmacological management of behavioral issues in the context of clinically relevant DDIs, complex dose titrations, and FFA AEs.

## 3 FACTORS TO CONSIDER

### 3.1 Managing FFA treatment with concomitant ASMs

When treating seizures associated with DS or LGS, polypharmacy is often needed to manage different seizure types. This increases the likelihood of DDIs and may lead to worsening cognition and/or sleep due to AEs from ASMs.^11^, ^20^, ^55^ In addition to FFA, we focus on DDIs and cognitive AEs associated with the range of ASMs the four patient cases were prescribed (VPA, STP, CLB, oxcarbazepine [OXC]) and/or those licensed for both DS and LGS (including CBD).

#### 3.1.1 Drug–drug interactions

The most prevalent site of pharmacokinetic interactions is that involving hepatic metabolism. Since many ASMs are metabolized via cytochrome p450 (CYP) and/or uridine glucuronyl transferases (UGTs), they are susceptible to enzyme induction or inhibition.^56^

FFA is metabolized to its active metabolite norfenfluramine (norFFA) by CYP1A2, CYP2B6, and CYP2D6^25^, ^57^, ^58^; FFA dose adjustments may therefore be needed when co‐administered with strong enzyme inhibitors or inducers, like STP.^25^, ^58^ The maximum FFA dose when patients are on concomitant STP is 0.4 mg/kg/day (total daily maximum = 17 mg), versus 0.7 mg/kg/day (or 26 mg/day) without concomitant STP.^59^ While CBD is a weak CYP1A2 inhibitor^60^ and thus may increase FFA and reduce norFFA levels, there is no real‐world evidence to suggest FFA requires a dose adjustment when co‐administered with CBD. In vitro studies found that FFA is unlikely to cause increases or decreases of serum levels of other ASMs^61^; therefore, dose adjustment of other ASMs, including CBD, is not needed when co‐administered with FFA.^62^

On the other hand, older generation ASMs are more likely to have DDIs, primarily due to their effect on the CYP enzyme system.^11^ This is particularly an issue with VPA formulations. VPA is a broad‐spectrum, frequently used ASM due to experience, cost effectiveness, and the added advantage of acting as a mood stabilizer.^22^ It is, however, extensively metabolized by the CYP system (CYP2A6, CYP2C9, CYP2C19, CYP2B6) and UGT (UGT1A3, UGT2B7).^56^ VPA and its derivatives are also broad‐spectrum inhibitors of CYP enzymes, resulting in decreased clearance and increased likelihood of substrate AEs, such as LTG and rufinamide (RUF), also extensively used for LGS.^11^, ^20^, ^24^, ^63^ Since VPA is a mainstay first‐line ASM in DS and LGS (Figure 1; Table 1)^3^, ^4^, ^10^, ^13^, ^20^, ^22^, ^24^ these DDIs are important to consider. There were, however, no clinically relevant DDIs associated with VPA in the four patients described.

STP is a potent inhibitor of CYP3A4, CYP1A2, CYP2B6, CYP2C8, and CYP2C19, which may require dose reduction of certain ASMs when concomitantly administered (eg, CLB or FFA).^56^, ^62^, ^64^ Since CLB should be given concomitantly with STP, CLB dose reduction in the setting of increased sedation or somnolence is recommended.^64^ When managing DS in clinical practice and wishing to reduce ASM burden, STP is often discontinued. As for Case 2, gradually weaning STP was suggested with FFA initiation, which decreased the need for a lower FFA maximum daily dose (17 mg/day). In real‐world studies of patients with DS and LGS, STP was the most frequently discontinued ASM after starting FFA^65^, ^66^, ^67^; bromide was also stopped or reduced in one German study.^65^

CLB was used in the three patients with DS. CLB is a CYP3A4 and 2C19 substrate and, therefore, its metabolism is affected by inducers or inhibitors; co‐administration with inhibitors (eg, felbamate or CBD) may increase CLB efficacy but also cause an increase in AEs, specifically somnolence and sedation. Therefore, CLB dose reductions are often warranted.^56^, ^60^, ^68^

CBD is approved for seizures associated with DS and LGS (and tuberous sclerosis, TSC) in the US and EU and is metabolized by CYP2C19 and CYP3A4.^24^, ^60^ It is associated with bi‐directional interactions with CLB, which may contribute to efficacy in seizure reduction but worsening of AEs. As such, the dose of CLB is often reduced when administered concomitantly with CBD. An increased dose of CBD may be required if co‐administered with strong enzyme inducers. Also important is the need for baseline and frequent liver enzyme monitoring in patients taking concomitant VPA, as transaminase elevations may occur.^60^

OXC is also associated with pharmacokinetic DDIs, as it is a weak CYP2C19 inhibitor which may increase VPA concentrations when administered concomitantly. This was not observed in Case 4, nor is it typically observed clinically in individuals with epilepsy. OXC, like carbamazepine, is contraindicated in DS due to its sodium channel blocking action.^23^, ^62^ OXC is also not indicated in LGS, but if used, caution should be exercised as it may trigger drop attacks.^20^

Since many ASMs have a narrow therapeutic index, the use of therapeutic drug monitoring (TDM) can be considered when there is a question of efficacy, safety, or monitoring of DDIs.^62^, ^69^ For specific older ASMs (eg, VPA), the use of TDM is common, but for FFA, STP and CBD, TDM is not generally indicated. In the case of increased fatigue, somnolence, or concomitant use with STP, providers may choose to proactively reduce doses, but checking FFA concentrations can also be considered,^70^ although this is not commonly done in practice. When adding FFA to a bromide‐containing regimen, bromide levels should be checked, as concentrations may increase, and doses may need to be subsequently reduced. In general, clinicians need to be aware of the impact of starting and discontinuing ASMs, especially those that are associated with DDIs. When an enzyme inducer is stopped, there may be a rebound increase in serum levels of the remaining ASM, resulting in increased toxicity; conversely, if an inhibitor is to be discontinued abruptly, normal/increased metabolism may resume and the risk for seizure activity increases.

Beyond the scope of this paper is the important consideration that pharmacokinetics of ASMs may vary due to patient‐related factors, such as age and comorbidities.^11^, ^19^, ^23^ Additionally, pharmacodynamic interactions may cause antiseizure effects to be additive, synergistic, or antagonistic.^23^

Compared to other ASMs, FFA offers the advantage of not being associated with many clinically relevant DDIs. Additionally, in DS real‐world studies, treatment with FFA allowed for a reduction or discontinuation of concomitant ASMs in up to 51% of patients, thereby reducing polypharmacy and risk for DDIs and AEs.^65^, ^66^, ^71^ FFA has also been reported effective as monotherapy in a single case.^72^

#### 3.1.2 Cognition and sleep

ASM polytherapy is associated with an increased risk for cognitive AEs, including memory loss. In turn, this can lead to reduced QOL and treatment nonadherence.^73^, ^74^ The cases in this review did not experience any cognitive AEs that were clearly related to their current ASMs, noting that cognitive deficits are an integral part of their DEEs. A literature review evaluating ASMs commonly used to manage DEEs reported that VPA, CLB, and STP exert neutral effects on cognition, while CBD and FFA may have positive effects on cognition.^74^ Real‐world studies of caregiver‐ and clinician‐reported outcomes have highlighted nonseizure benefits with FFA, including improved cognition, focus, and alertness.^75^, ^76^ In the FFA clinical trial program, executive function (EF) was evaluated as part of the safety analysis, revealing no worsening on the Behavior Rating Inventory of Executive Function (BRIEF®). Moreover, various post‐hoc analyses of the DS and LGS clinical trials^77^, ^78^, ^79^, ^80^ have demonstrated improvements in aspects of everyday EF, including in adult patients with LGS.^81^, ^82^ Streamlining ASM regimens and reducing polypharmacy could also mitigate cognitive regression.

Of the cases presented, only Case 4 experienced sleep issues, likely due to nocturnal seizure activity. This is common with DEEs, as there is a bidirectional relationship between seizure activity and sleep; seizure activity impacts sleep and poor sleep quality can provoke seizures.^74^, ^83^ In adults with DS, the predominant seizure pattern is brief nocturnal tonic–clonic seizures,^84^ and in LGS, sleep dysfunction may be secondary to nocturnal tonic seizures.^85^, ^86^ Sleep disturbances can also contribute to behavioral issues and decreased QOL, further complicated by polypharmacy.^20^, ^74^, ^86^, ^87^ Some ASMs are associated with excessive daytime sleepiness and insomnia and medications used to treat sleep issues may aggravate seizures.^74^, ^86^ FFA may be associated with somnolence and lethargy,^25^ but caregivers and clinicians of patients with DS have reported improvement in sleep quality when treated with FFA.^75^, ^76^ While this was reported to be a nonseizure benefit in DS, Case 4 with LGS exhibited improvement in sleep potentially due to decreased seizure activity after starting FFA.

These nonseizure outcomes are also reflected in improvements in Clinical Global Impression of Improvement (CGI‐I) scores as rated by investigators and caregivers in the DS and LGS RCTs and open‐label extension (OLE) studies.^16^, ^33^, ^34^, ^35^, ^37^, ^53^ Real‐world studies have also demonstrated improvements in CGI‐I in patients with DS and LGS treated with FFA, including but not limited to improvements in behavior, communication, and motor skills.^65^, ^66^, ^67^, ^71^

**Practical considerations for managing FFA with concomitant ASMs**

There is no one‐size‐fits‐all approach for treating DS or LGS, as evidenced by the heterogeneity in the seizure types and ASM regimens in the cases described. Experts agree that in general, no more than 3 ASMs should be used, which aligns with published recommendations. Lessening polypharmacy in patients on ASMs can reduce the likelihood of cognitive AEs, sleep issues, and potential for DDIs. Other than a dose reduction required when concomitantly administered with STP, FFA is associated with fewer clinically relevant DDIs than most ASMs. Additionally, patients treated with long‐term FFA have not demonstrated worsening of cognition and may experience improvements in sleep and subsets of EF.

### 3.2 Managing behavioral comorbidities

Neurodevelopmental comorbidities are a key part of all DEEs.^6^, ^74^ Specifically, as with Cases 2, 3, and 4, comorbidities such as depression, anxiety, aggression, attention deficit with hyperactivity, and autism spectrum disorders may be observed in patients with DS or LGS.^4^, ^11^, ^13^, ^17^, ^19^, ^22^, ^74^, ^88^ In a caregiver survey by Lagae et al, patients with DS were experiencing an average of 4 identifiable behavioral impairments or comorbidities.^17^ A 10‐year follow‐up study of patients with DS identified that, together with epilepsy severity, increased behavioral difficulties were significant predictors of lower health‐related QOL.^89^

Optimizing seizure outcomes is important, but tailoring therapy to improve behavioral symptoms that seriously impact QOL and adversely affect long‐term functioning is also imperative.^4^, ^11^, ^12^, ^13^, ^22^ Unfortunately, there are multiple challenges involved in managing behavioral issues pharmacologically – some ASMs may exacerbate or cause behavioral AEs,^4^, ^13^ and many antipsychotics and antidepressants may interact with ASMs.^56^ These complexities contribute to behavioral issues often being under‐treated. Out of 584 unique responses from caregivers of patients with DS in Europe (mean age, 10.6 years), only 6% of patients took antipsychotic medication for management of behavioral comorbidities and only 6% were on medication for attention deficit hyperactivity disorder.^17^

As with the cognitive AEs described earlier, VPA, CBD, and FFA are generally neutral or positive when it comes to behavioral effects. STP, however, has been associated with behavioral changes.^74^ In practice, CLB causes irritability and aggression in more than the ~10% of cases reported in the literature.^68^, ^74^

ASMs that interact with CYP substrates are likely to be problematic, since many neuroleptic agents are metabolized by CYP450.^62^ Antipsychotics metabolized by CYP450 include: risperidone, aripiprazole, clozapine, olanzapine, and quetiapine. Risperidone is extensively metabolized by CYP2D6 and aripiprazole is a CYP3A4 substrate, making them both susceptible to increased or decreased plasma concentrations depending on concomitant administration of a CYP inhibitor or inducer, respectively.^90^ Some antidepressants are also metabolized via CYP450, such as fluvoxamine, citalopram, paroxetine, and sertraline.^91^ Their therapeutic and toxic effects could be enhanced or reduced by inhibitors or inducers, respectively.^91^ Sertraline, fluoxetine, and paroxetine inhibit CYP2D6, potentially enhancing antiseizure activity or AEs of certain ASMs.^91^

While FFA does not appear to have as many clinically relevant DDIs with CYP450 substrates as other ASMs (see 3.1.1), use within 14 days of monoamine oxidase inhibitors (MAOIs) is contraindicated, and it is advisable to use caution with other serotonergic medications due to the potential increased risk for serotonin syndrome.^23^, ^59^ Since many neuroleptic agents work via serotonergic mechanisms, it is recommended to ensure careful observation during treatment initiation and dose increases. Prior to initiating FFA, Case 3 was being managed with risperidone. To minimize the risk of serotonin syndrome, most clinicians would adapt treatment accordingly. In this case, the switch to aripiprazole (which exhibits less serotonergic potential) was suggested and an improvement in behavior was observed. More data and clinical experience are needed to understand the risk of FFA with antipsychotics and/or antidepressants.

Post‐ictal states are often reduced with improved seizure control. Patients may thus have increased periods of alertness, which can, in turn, expose behavioral issues.^19^ It is also possible, however, that an improvement in behavior may occur secondary to better seizure control, as seen with Case 4. The caregiver‐ and clinician‐reported outcomes studies by Jensen et al revealed that FFA treatment was associated with shorter post‐ictal recovery times,^75^, ^76^ but there has been no evidence of worsening behavioral issues in studies of FFA. In fact, the international DS consensus panel of physicians and caregivers indicated that patients on FFA demonstrated “improved alertness and/or behavior”.^3^

Behavioral issues in adults with DEEs should be managed by adult neurologists, ideally with psychiatry and psychology input. There is still an unmet need for a simple tool or scale to evaluate nonseizure outcomes in patients with DEEs.

**Practical considerations for managing behavioral comorbidities while on FFA**

Impact on management of behavioral comorbidities should be considered when initiating ASMs, including potential for DDIs with neuroleptic medications. MAOIs must be avoided within 14 days of FFA, and caution should be used with other serotonergic agents. FFA has been associated with shorter post‐ictal recovery and no evidence of worsening behavior.

### 3.3 FFA dose titration and responsiveness

In general, initiation and titration of ASMs are associated with the “start low, go slow” approach to minimize AEs and allow patients to reach optimal, lowest effective, maintenance doses.^92^ In line with the protocols of the RCTs, the FINTEPLA® prescribing information recommends a starting dosage of FFA 0.2 mg/kg/day. FFA titrations are then recommended to be performed weekly, but a more rapid titration can be implemented (every 4 days) if a patient is not on concomitant STP and it aligns with caregiver/patient goals.^25^

Three of the patients began FFA treatment at 0.2 mg/kg/day; Case 2 was initiated at a lower dose due to the concomitant use of STP and weight of 88 kg. Titrations for all the patients varied over time (Table 3). In Case 1, the dose was increased after 2 weeks, and subsequent titrations occurred every 1–2 weeks. Case 4 was part of the open‐label LGS study^54^ and, thus, was managed per protocol, which reflected a slower titration than is generally recommended; Case 2 was also titrated slowly. In practice, titrating doses every 2 weeks seems to generally be ideal and should not be performed more frequently than weekly (as indicated in the prescribing information)^25^; this allows prescribers and caregivers to adequately assess impact of FFA on seizure frequency and any AEs that might emerge. Over the past 7 years, Case 4's dosage has decreased and she has remained at 0.29 mg/kg/day.

**TABLE 3:** Fenfluramine titrations in four cases.

Starting FFA Dose	Case 1^a^	Case 2	Case 3^a^	Case 4
	0.2 mg/kg/day	<0.2 mg/kg/day	0.2 mg/kg/day	0.2 mg/kg/day
Titrations	Increased by 0.05 mg/kg/day every 1–2 weeks until current dose	Increased from 0.15 mg/kg/day to 0.2 mg/kg/day (over 8 months), then up to 0.25 mg/kg/day (over 7 months)	Initiated therapy at capped dose and was titrated down to the current maintenance dose	Per protocol – increased to 0.4 mg/kg/day after 1 month, then increased to 0.5 mg/kg/day after Month 2 Subsequent titrations over 7 years
Final/Current FFA Dose	0.35 mg/kg/day	0.25 mg/kg/day	0.19 mg/kg/day	0.29 mg/kg/day

Abbreviation: FFA, fenfluramine.

^a^ Patient on concomitant stiripentol.

The goal of FFA titration, as with any ASM, is to find the minimally effective dose by Week 6–8 of treatment. Experts agreed that most patients are adequately managed at around 0.4 mg/kg/day; in all 4 case examples, patients were on <0.4 mg/kg/day even without concomitant STP treatment (Table 3). Careful attention to concomitant medications is needed and anticipatory adjustments should be made if a clinically relevant DDI is expected.

Another factor to consider with the use of FFA is the impact of age and weight. As patients gain weight and/or age increases, they will reach the capped daily dose (17 mg with STP or 26 mg without STP) before achieving the maximum weight‐based dosage (0.4 mg/kg/day or 0.7 mg/kg/day); this, however, does not impact clinical outcomes. A recent post‐hoc analysis of patients in the LGS RCT and OLE studies indicated that there was no difference in frequency of seizures associated with a fall in patients treated with FFA who were dose‐capped (≥37 kg) versus those that continued to receive weight‐based treatment (<37 kg).^93^ Experts noted that they found the most benefit in up‐titrating adults every 2 weeks and modifying doses based on volume (mL) rather than strength or dose, as they would with children. One expert noted he generally titrates by 1 mL/day weekly.

ASM response should evaluate the ability to achieve improvements in both seizure and nonseizure outcomes. Adequate trials depend on the ASM half‐life, speed of titration, dose attained, and patient/caregiver goals. Generally, an FFA trial should last at least 3 months, but experts suggest that in some patients a shorter trial (eg, 1 month) can be considered. After initiating FFA, in the DS clinical trials, reduction in convulsive seizure frequency was observed within 3–4 weeks, and drop seizure frequency reduction was noted within 2 weeks in the LGS RCT.^25^

Reduction of the most problematic seizure type(s) is often prioritized.^14^ In DS, control of convulsive seizures is usually prioritized over nonconvulsive seizures,^3^ as in all epilepsy syndromes a higher rate of convulsive seizures is associated with an increased risk of sudden unexpected death in epilepsy (SUDEP).^11^, ^94^, ^95^, ^96^, ^97^ There is data to support efficacy in GTCS reduction in patients with DS and LGS.^33^, ^39^ Cross et al identified that SUDEP mortality rates in patients with DS treated with FFA were substantially lower than in published historical controls.^98^, ^99^

**Practical considerations for titrating and assessing FFA**

FFA dose titrations must be individualized, taking into consideration concomitant medications, patient weight, and the DEE. Titrations every 2 weeks may be ideal and an adequate trial of at least 1–3 months is recommended.

### 3.4 FFA safety and tolerability

#### 3.4.1 Adverse events

Among the 4 cases described, only one patient reported an AE secondary to treatment with FFA. Case 1 experienced a weight loss of 1.1 kg after FFA was initiated; this patient did not tolerate management with high‐calorie shakes but, after VPA dose was decreased, his weight loss stabilized and he continues on FFA treatment.

While underlying DEEs may impact weight and growth, many ASMs also affect appetite and weight gain; this requires careful monitoring of patient weight during treatment.^24^, ^100^ Specifically, ASMs associated with decreased appetite and/or weight loss include CBD, brivaracetam, topiramate, bromide, felbamate, RUF, ethosuximide, zonisamide, STP, and FFA.^24^, ^100^ In the OLE study of FFA in DS, the most common treatment‐emergent AEs reported were pyrexia, nasopharyngitis, decreased blood glucose, and decreased appetite.^36^ In the LGS OLE of FFA, fatigue and decreased appetite were the most commonly reported AEs.^16^ Relative to a historical control population of patients with DS, patients on long‐term (≥12 months) treatment with FFA (*N* = 279) demonstrated minimal impact on height or weight over time and there were no substantial dose‐dependent changes observed from the baseline at Month 12 nor Month 24.^101^ To manage decreased appetite and/or weight loss, dietary interventions or adjustments to other ASMs can be implemented.^101^ As with Case 2, where STP was discontinued in parallel with FFA initiation, experts recommend dose reduction or discontinuation of other ASMs that may affect appetite, to improve tolerability of FFA.

It is important to note that FFA is associated with a risk of valvular heart disease and pulmonary arterial hypertension (VHD/PAH)^25^ due to cases that occurred when FFA was marketed as an anorectic agent, but thus far in the clinical program there have been no reports of VHD or PAH.^36^, ^102^, ^103^, ^104^ As of June 24, 2023, post‐authorization (nonclinical trial) exposure to FFA is estimated at 5203.2 patient‐years globally, and post‐marketing reports have identified one probable case of PAH in a patient with DS which could be associated with FFA. In this patient, echocardiogram (ECHO) revealed a pulmonary arterial systolic pressure (PASP) of 35 mmHg as well as other features compatible with PAH which led to discontinuation of FFA. A follow‐up ECHO revealed no elevation in PASP and other features suggestive of PAH also improved.^105^

FFA distribution is restricted in the US, via the FINTEPLA® Risk Evaluation and Mitigation Strategy (REMS) program, and in the EU, via the Controlled Access Program (CAP). These programs require regular ECHO monitoring to ensure any cardiac abnormalities will be identified before a patient becomes symptomatic or progresses to VHD or PAH; FFA can then be discontinued. The experts agree that the benefits associated with FFA use outweigh the potential cardiac risk. Overall, FFA is regarded by caregivers and physicians as a therapy with “good tolerability”.^3^

#### 3.4.2 Use of ASMs in females of child‐bearing age

As DS and LGS are both chronic conditions that persist into adulthood, one important factor to consider as females approach child‐bearing age is the possibility of reproduction, acknowledging that most women will not reproduce due to the severity of their disease. In the rare cases where a pregnancy occurs, the impact that their DEE and ASM regimen would have on the pregnancy and baby must be considered. Epilepsy and the use of ASMs are associated with pregnancy complications, fetal growth restriction, and congenital malformations,^106^ thus most clinicians caring for patients with DS or LGS would not favor reproduction. Perinatal counseling that strongly emphasizes contraception and addresses risks involving the underlying DEE and ASM use is strongly encouraged.

All the major pregnancy registries have identified that VPA and its derivatives have the highest risk of major congenital malformations of all first generation ASMs, a risk that is dose‐dependent.^106^ There is also evidence that VPA use throughout the pregnancy is associated with autism spectrum disorder and attention deficit with hyperactivity in the child,^107^ as well as poor cognitive outcomes including reduced IQ.^106^, ^107^, ^108^ Hypospadias have been reported with OXC^109^ but some sources consider OXC at low risk for anomalies.^110^ There is sparse data on the use of CLB monotherapy since it is typically used as adjunctive treatment,^109^ but animal studies have shown developmental toxicity and fetal malformations.^68^ Similarly, there are currently no data on the use of CBD, STP, or FFA in pregnant women, but, as with other ASMs, animal data revealed fetal harm, including growth impairment in all three and fetal malformation in STP and FFA.^25^, ^60^, ^64^ As with CLB, CBD, and STP, women who become pregnant while taking FFA are encouraged to enroll in a pregnancy registry that tracks pregnancy outcomes in large cohorts.

In the cases reviewed here, one patient was of reproductive age (Case 4), but caregivers and clinicians involved in her care agreed to continue all treatments, including VPA.

**Practical considerations for managing FFA AEs and perinatal counseling**

As with many ASMs, it is important to monitor weight loss in patients treated with FFA. While on FFA, reducing or discontinuing concomitant ASMs may help to manage loss of appetite or weight loss. Regular ECHO monitoring while on FFA treatment allows the mitigation of any potential cardiovascular risk.

For females of child‐bearing age with an underlying DEE, perinatal counseling involves an emphasis on contraception and transparency surrounding ASM risks. Data surrounding newer ASMs (eg, FFA, STP, CBD) are needed, and in the rare case of pregnancy, enrollment in a pregnancy registry is strongly encouraged.

### 3.5 Conclusions

The four case vignettes presented here demonstrate the clinical complexities clinicians face when managing patients with DEEs, such as DS and LGS. Polypharmacy, DDIs, managing behavioral comorbidities, dose titrations, and AEs that impact use are just a few of the issues that arise when ASMs are prescribed. As prescribers strive to optimize and finetune ASM regimens, there may be a significant impact on patient and caregiver QOL.

FFA has a unique mechanism of action, is generally well‐tolerated, is associated with a low risk of DDIs, and offers a broad‐spectrum profile of efficacy involving both seizure and nonseizure outcomes. In the DS and LGS clinical program, as well as real‐world data, FFA has demonstrated sustained reductions in convulsive seizure frequency, in particular GTCS reduction which may translate to a reduction in SUDEP. Clinically meaningful improvements in nonseizure outcomes have also been reported.

Incorporation of some of the considerations described here may inform prescribing practice and lead to improved patient and caregiver QOL. Future studies of FFA can focus on safety and effectiveness of capped doses in patients ≥37.5 kg, ability of FFA to reduce adjunctive ASM polytherapy, concomitant use of serotonergic agents, and the treatment of seizures in patients under 2 years old.

## AUTHOR CONTRIBUTIONS

ECW, LL, and AS provided cases for inclusion. All authors contributed to the conceptualization of the manuscript, were involved in critically revising the drafts, and approved the final version for publication.

## FUNDING INFORMATION

This work has been funded by UCB Pharma.

## CONFLICT OF INTEREST STATEMENT

**ECW** has received consulting income from Acadia, Amicus, Neurocrine and Encoded Therapeutics. She also receives income from Epilepsy.com for serving as Co‐Editor in Chief. **LL** has received grants, and is a consultant and/or speaker for Zogenix (now a part of UCB), LivaNova, UCB Pharma, Shire, Eisai, Novartis, Takeda/Ovid, NEL, and Epihunter. **IES** has served on scientific advisory boards for BioMarin, Chiesi, Eisai, Encoded Therapeutics, GlaxoSmithKline, Knopp Biosciences, Nutricia, Rogcon, Takeda Pharmaceuticals, UCB, Xenon Pharmaceuticals, Cerecin; has received speaker honoraria from GlaxoSmithKline, UCB, BioMarin, Biocodex, Chiesi, Liva Nova, Nutricia, Zuellig Pharma, Stoke Therapeutics, Eisai, Akumentis; has received funding for travel from UCB, Biocodex, GlaxoSmithKline, Biomarin, Encoded Therapeutics, Stoke Therapeutics and Eisai; has served as an investigator for Anavex Life Sciences, Cerevel Therapeutics, Eisai, Encoded Therapeutics, EpiMinder Inc, Epygenyx, ES‐Therapeutics, GW Pharma (now Jazz Pharmaceuticals), Marinus, Neurocrine BioSciences, Ovid Therapeutics, SK Life Science, Takeda Pharmaceuticals, UCB, Ultragenyx, Xenon Pharmaceuticals, Zogenix (now a part of UCB), and Zynerba; and has consulted for Care Beyond Diagnosis, Epilepsy Consortium, Atheneum Partners, Ovid Therapeutics, UCB, Zynerba Pharmaceuticals, BioMarin, Encoded Therapeutics and Biohaven Pharmaceuticals; and is a Non‐Executive Director of Bellberry Ltd and a Director of the Australian Academy of Health and Medical Sciences and the Royal Society (Australia). She may accrue future revenue on pending patent WO61/010176 (filed: 2008): Therapeutic Compound; has a patent for *SCN1A* testing held by Bionomics Inc and licensed to various diagnostic companies; has a patent molecular diagnostic/theranostic target for benign familial infantile epilepsy (BFIE) [PRRT2] 2011904493 & 2 012 900 190 and PCT/AU2012/001321 (TECH ID:2012–009). **JHC** has received research grants from Zogenix (now a part of UCB), Marinus, GW Pharma (now Jazz Pharmaceuticals), Vitaflo, Stoke Therapeutics, Ultragenyx, National Institute of Health Research (NIHR), EPSRC, GOSH Charity, ERUK, the Waterloo Foundation, and the Great Ormond Street Hospital NIHR Biomedical Research Centre; and has served as consultant/advisor for Zogenix (now a part of UCB), GW Pharma (now Jazz Pharmaceuticals), and Biocodex for which remuneration was made to the department, outside of the submitted work; serves as Chair of the Medical Board for Dravet UK, Hope for Hypothalamic Hamartoma, and Matthews Friends and endowed chair at UCL Great Ormond Street Institute of Child Health. **NS** has served on scientific advisory boards for GW Pharma (now Jazz Pharmaceuticals), BioMarin, Arvelle, Marinus and Takeda; has received speaker honoraria from Eisai, Biomarin, Livanova, Sanofi; and has served as an investigator for Zogenix (now a part of UCB), Marinus, Biomarin, UCB and Roche. **AS** reports personal fees and grants from Angelini Pharma, Biocodex, Desitin Arzneimittel, Eisai, Jazz (GW) Pharmaceuticals, Marinus Pharmaceuticals, Medtronic, Takeda, UCB Pharma (Zogenix), and UNEEG. We confirm that we have read the Journal‘s position on issues involved in ethical publication and affirm that this report is consistent with those guidelines.

## ETHICS STATEMENT

Informed consent was obtained from the patients, or parents or legal representatives of the involved patients.

## Data Availability

Data from noninterventional studies are outside of UCB's data sharing policy and are unavailable for sharing.
